# Retrospective comparison of long-term functionality and revision rate of two different shunt valves in pediatric and adult patients

**DOI:** 10.1007/s00701-023-05719-y

**Published:** 2023-08-02

**Authors:** Lewin-Caspar Busse, Daniel Dubinski, Florian Gessler, Nazife Dinc, Jürgen Konczalla, Marcus Czabanka, Christian Senft, Thomas M. Freiman, Peter Baumgarten

**Affiliations:** 1 Department of Neurosurgery, University Hospital Frankfurt, Goethe University, Frankfurt, Germany; 2 Department of Neurosurgery, University Medicine Rostock, Rostock, Germany; 3 Department of Neurosurgery, University Hospital Jena, Friedrich Schiller University, Jena, Germany

**Keywords:** Hydrocephalus, Cerebrospinal fluid, Shunt valve, Revision, proGAV, Child

## Abstract

**Purpose:**

The most frequent therapy of hydrocephalus is implantation of ventriculoperitoneal shunts for diverting cerebrospinal into the peritoneal cavity. We compared two adjustable valves, proGAV and proGAV 2.0, for complications resulting in revision surgery.

**Methods:**

Four hundred patients undergoing primary shunt implantation between 2014 and 2020 were analyzed for overall revision rate, 1-year revision rate, and revision-free survival observing patient age, sex, etiology of hydrocephalus, implantation site, prior diversion of cerebrospinal fluid, and cause of revision.

**Results:**

All data were available of all 400 patients (female/male 208/192). Overall, 99 patients underwent revision surgery after primary implantation. proGAV valve was implanted in 283 patients, and proGAV 2.0 valves were implanted in 117 patients. There was no significant difference between the two shunt valves concerning revision rate (*p* = 0.8069), 1-year revision rate (*p* = 0.9077), revision-free survival (*p* = 0.6921), and overall survival (*p* = 0.3232). Regarding 1-year revision rate, we observed no significant difference between the two shunt valves in pediatric patients (40.7% vs 27.6%; *p* = 0.2247). Revision operation had to be performed more frequently in pediatric patients (46.6% vs 24.8%; *p* = 0.0093) with a significant higher number of total revisions with proGAV than proGAV 2.0 (33 of 59 implanted shunts [55.9%] vs. 8 of 29 implanted shunts [27.6%]; *p* = 0.0110) most likely due to longer follow-up in the proGAV-group. For this reason, we clearly put emphasis on analyzing results regarding 1-year revision rate.

**Conclusion:**

According to the target variables we analyzed, aside from lifetime revision rate in pediatric patients, there is no significant difference between the two shunt valves.

## Introduction

Hydrocephalus is a common and often complex disease. In most of the patients, hydrocephalus leads to a progressive ventricular dilatation [34]. If left untreated, hydrocephalus leads to severe patient impairment or even death [14]. Implantation of ventriculoperitoneal shunt systems with adjustable shunt valves represents a common treatment for both pediatric and adult patients [1, 2, 7, 15, 17]. Implantation of shunt systems can lead to a variety of complications, including shunt infection, shunt obstruction or disconnection, tubing migration, and overdrainage or underdrainage of cerebrospinal fluid due to valve malfunction [17]. Several studies have already addressed risk factors, rate of shunt revision, and possibilities for prevention [1, 17, 18, 26]. However, these complications depend on the age and sex of the patient, etiology of hydrocephalus, underlying diseases, and whether the shunt was implanted in primary (first shunt implantation), secondary (second shunt implantation), tertiary (third shunt implantation), or even quaternary (fourth shunt implantation) situations [4]. In particular, pediatric patients and common afflictions such as intraventricular hemorrhage of preterm infants significantly affect shunt failure- and complication rates [3, 5, 6, 11, 22, 31, 35]. But also in elderly patients where hydrocephalus is seen in the posthemorrhagic- or idiopathic normal pressure variant, it should not be left unconsidered while looking at rates of shunt revision [20]. However, adjustable valves such as the Miethke proGAV (Aesculap, Miethke, Potsdam, Germany) are still in great demand and contribute to minimizing the number of surgical revisions [2]. As a result of progressive development of these shunt valves, our institutions have largely replaced the proGAV by the proGAV 2.0 valve. The new proGAV 2.0 was completely mechanically reconstructed, now consisting of a tactile adjustable chamber, facilitating the pressure adjustment for the physician. Since the more complex structure of the new valve, it potentially bears the risk of a higher failure rate. To date, however, in vivo there are only few studies comparing these two shunt valves regarding patient characteristics. The aim of our monocentric study was to look for differences in the two shunt systems regarding shunt failure, including other potential and known risk factors for shunt failures in patients with ventriculo-peritoneal shunts.

## Materials and methods

### Patients

A total of 453 patients who underwent shunt surgery between 2014 and 2020 were retrospectively evaluated. Inclusion criteria were implantation of a Miethke proGAV or proGAV 2.0 valves, while implantation of other devices or surgery without implanting a valve was excluded. The cohort of patients in primary situation consisted of 88 pediatric patients as defined by a patient age of < 18 years, 194 adult patients between the age of 18 and 64, and 118 elderly patients with an age of ≥ 65 years. Mean age of patients included in this study was 46.2 years and 208 patients were (52%) were women.

Regarding revision surgery, we analyzed associations regarding the etiology of hydrocephalus including primary vs. secondary hydrocephalus, malresorptivus vs. occlusus, frequent diseases such as subarachnoid hemorrhage, normal pressure hydrocephalus, intraventricular hemorrhage, congenital aqueductal stenosis, intracerebral hemorrhage and glioblastoma, localization of the shunt catheter, and prior diversion of cerebrospinal fluid. Categorization of etiology was performed choosing the underlying disease which is most likely to cause the hydrocephalus. In the primary situation, implementation of the shunt catheter was routinely performed in the frontal area of the right lateral ventricle.

The study was conducted in accordance with the Declaration of Helsinki. The study was approved by the local ethics committee of the Goethe University of Frankfurt, EC number 20–995, accepted December 28th, 2020. We confirm that we have read the Journal’s position on issues involved in ethical publication and affirm that this report is consistent with those guidelines.

### Valves

From 2014 to 2017, mostly proGAV valves were used when performing shunt surgery. In 2017, our facility nearly completely replaced the proGAV valve by the proGAV 2.0 valve. As per this switch, there was no algorithm for choosing either the proGAV or the proGAV 2.0 valve.

### Evaluation of clinical data

Patient clinical data were retrospectively extracted from the electronic patient records. These records included surgery reports, physician’s letters, radiological diagnostics, anesthesiologic protocols, and microbiological screening reports.

### Statistical analysis

Statistical analysis and figure editing were performed using JMP 14.0 software (SAS Institute, Cary, NC, USA) and the open-source GIMP2 program. Descriptive statistical methods mean (± SD) or median and interquartile range (IQR) (25–75%) were used to analyze data.

Survival analyses were performed using Kaplan–Meier method with Wilcoxon and log-rank test. For univariate analysis, we used likelihood-ratio and chi-square tests for categorial variables. Collinearity between the variables in multivariate analyses was tested by Spearman’s rank correlation coefficient. A Rho of < 0.9 was seen as non-critical. We applied logarithmic regression for the categorial variables (1-year revision rate) as multivariate analysis. Cox proportional hazards were used for multivariate analysis of the overall revision rate. A significance level of alpha < 0.05 was chosen for all tests. We included patient age, gender, implanted shunt system, etiology of hydrocephalus, localization of ventricle catheter, and prior CSF diversion. To evaluate which factors were associated with a higher number of shunt revisions and complications, we analyzed revision rate, 1-year revision rate, revision-free survival (RFS), and overall survival (OAS).

Moreover, in our analysis, we distinguished between 1-year revision rate in primary situation for all patients (Table 1) and pediatric patients (Table 2) and lifetime revision rate in primary situation for all patients (Table 3) and pediatric patients (Table 4).

In addition, we performed Proportional-Hazards analysis regarding the factors “pediatric,” “elderly,” and “intraventricular hemorrhage” (Table 5).

### Follow-up protocol

Revision-free shunt survival was defined as a follow-up period without an event of any kind of shunt revision surgery.

In pediatric patients presenting an uneventful clinical course, a radiological examination of the shunt course was carried out immediately after the operation using an X-ray of the abdomen and an MRI of the head, if possible, ultrasound of the head, and an MRI scan once a year. This was done until the pediatric patients reached the age of majority.

For adults, a CT scan of the head and biplanar X-ray of the abdomen were performed immediately after the surgical procedure to assess the course of the shunt. If there were no relevant clinical events, no standard follow-up control was conducted in the further process and patients were released from follow-up after three uneventful months. Nevertheless, some of the patients had a longer follow-up time due to their underlying disease, which required regular follow-up controls.

## Results

### Patient cohort

The analyzed cohort of 453 patients underwent 530 surgeries in primary (400), secondary (51), tertiary (56), or quaternary (23) situations with an implantation of a proGAV or proGAV 2.0 valve in our neurosurgical facility. Of those patients, 400 underwent primary implantation of one of the two shunt valves named above (female/male 208/192) with an allocation of 88 pediatric patients (= 22%), 194 adult patients younger than 65 years (= 48.5%), and 118 elderly patients (= 29.5%). There was no significant difference in any of our target variables regarding patient sex (Likelihood-ratio: *p* = 0.5653).

Regarding the fact that there were no significant results in the univariate analysis of patients in secondary (data not shown), tertiary (data not shown), or quaternary situation (data not shown), we further analyzed the patient cohort in the primary surgical situation (Tables 1, 2, 3, 4, and 5).

**Table 1:** Distribution of shunt revisions within 1 year of initial shunt implementation

Parameter	*n*	No revision	Revision	Univariate analysis (likelihood/Pearson)	Multivariate analysis (logarithmic regression)
		Total (%)	Total (%)		
	400	316 (79.0)	84 (21.0)		
Sex				0.8673/0.8673	
Male	192	151 (78.6)	41 (21.4)		
Female	208	165 (79.3)	43 (20.7)		
Age					
Pediatric	88	56 (63.6)	32 (36.4)	< 0.0001/ < 0.0001	0.0085
Elderly	118	98 (83.1)	20 (16.9)	0.1912/0.1982	
Adult Other	194	162 (83.5)	32 (16.5)		
Shunt system				0.9077/0.9076	
proGAV	283	224 (79.2)	59 (20.8)		
proGAV 2.0	117	92 (78.6)	25 (21.4)		
Etiology of hydrocephalus					
Primary/secondary				0.7613/0.7598	
Primary	67	52 (77.6)	15 (22.4)		
Secondary	333	264 (79.3)	69 (20.7)		
Type of hydrocephalus				0.2538/0.2499	
*H. occlusus*	136	103 (75.7)	33 (24.3)		
*H. malresorptivus*	264	213 (80.7)	51 (19.3)		
Frequent diseases					
SAH	107	87 (81.3)	20 (18.7)	0.4894/0.4834	
NPH	53	41 (77.4)	12 (22.6)	0.7546/0.7528	
IVH	24	15 (62.5)	9 (37.5)	0.0556/0.0407	0.2344
CAS	19	17 (89.5)	2 (10.5)	0.2157/0.2508	
ICH	17	12 (70.6)	5 (29.4)	0.4031/0.3842	
Glioblastoma	11	8 (72.7)	3 (27.3)	0.6158/0.6045	
IIH	10	10 (100.0)	0 (0.0)	0.0288/0.0987	0.0353
Other	159				
Localization					
Left ventricle	84	72 (85.7)	12 (14.3)	0.1196/0.1305	
Right ventricle	304	234 (77.0)	70 (23.0)	0.0328/0.0396	0.0219
Bilateral	12	10 (83.3)	2 (16.7)	0.3479/0.3871	
Prior CSF diversion				0.0005/ < 0.0001	0.0340
EVD	170	138 (81.2) 13	32 (18.8)		
LD	15	(86.7)	2 (13.3)		0.0422
Ommaya reservoir	18	6 (33.3)	12 (66.7)		
No prior diversion	191	156 (81.7)	35 (18.3)		
Inconclusive	6				

*H. malresorp*, hydrocephalus malresorptivus; *SAH*, subarachnoid hemorrhage; *NPH*, normal pressure hydrocephalus; *IVH*, intraventricular hemorrhage; *CAS*, congenital aqueductal stenosis; *ICH*, intracranial hemorrhage; *IIH*, idiopathic intracranial hypertension; *CSF*, cerebrospinal fluid; *EVD*, external ventricular drain; *LD*, lumbar drain

**Table 2:** Distribution of shunt revisions of pediatric patients within 1 year of initial shunt implementation related to shunt system

Parameter	*n*	No revision	Revision	Univariate analysis (likelihood/Pearson)
		Total (%)	Total (%)	
	88	56 (63.6)	32 (36.4)	
Shunt system				0.2247/0.2301
proGAV	59	35 (59.3)	24 (40.7)	
proGAV 2.0	29	21 (72.4)	8 (27.6)

**Table 3:** Distribution of all shunt revisions

Parameter	*n*	No revision	Revision	Univariate analysis (likelihood/Pearson)	
		Total (%)	Total (%)		
	400	301 (75.2)	99 (24.8)		
Sex				0.5653/0.5652	
Male	192	142 (74.0)	50 (26.0)		
Female	208	159 (76.4)	49 (23.6)		
Age					
Pediatric	88	47 (53.4)	41 (46.6)	< 0.0001/ < 0.0001	
Elderly	118	97 (82.2)	21 (17.8)	0.0330/0.0371	
Adult	194	157 (80.9)	37 (19.1)		
Shunt system				0.8069/0.8073	
proGAV	283	212 (74.9)	71 (25.1)		
proGAV 2.0	117	89 (76.1)	28 (23.9)		
Etiology of hydrocephalus					
Primary/secondary				0.8972/0.8969	
Primary	67	50 (75.0)	17 (25.0)		
Secondary	333	251 (72.1)	82 (27.9)		
Type of hydrocephalus				0.2915/0.2885	
*H. occlusus*	136	98 (72.1)	38 (27.9)		
*H. malresorptivus*	264	203 (76.9)	61 (23.1)		
Frequent diseases					
SAH	107	84 (78.5)	23 (21.5)	0.3572/0.3620	
NPH	53	41 (77.4)	12 (22.6)	0.7001/0.7025	
IVH	24	13 (54.2)	11 (45.8)	0.0204/0.0136	
CAS	19	13 (68.4)	6 (31.6)	0.4910/0.4797	
ICH	17	12 (70.6)	5 (29.4)	0.6550/0.6490	
Glioblastoma	11	8 (72.7)	3 (27.3)	0.8458/0.8441	
IIH	10	8 (80.0)	2 (20.0)	0.7183/0.7245	
Other	159				
Localization					
Left ventricle	84	72 (85.7)	12 (14.3)		0.1271/0.1361
Right ventricle	304	222 (73.0)	82 (27.0)		0.0598/0.0667
Bilateral	12	10 (83.3)	2 (16.7)		0.7183/0.7245
Prior CSF diversion					< 0.0001/ < 0.0001
EVD	170	132 (77.6)	38 (22.4)		
LD	15	13 (86.7)	2 (13.3)		
Ommaya reservoir	18	4 (22.2)	14 (77.8)		
No prior diversion	191	149 (78.0)	42 (22.0)		
Inconclusive	6				

*H. malresorp*, hydrocephalus malresorptivus; *SAH*, subarachnoid hemorrhage; *NPH*, normal pressure hydrocephalus; *IVH*, intraventricular hemorrhage; *CAS*, congenital aqueductal stenosis; *ICH*, intracranial hemorrhage; *IIH*, idiopathic intracranial hypertension; *CSF*, cerebrospinal fluid; *EVD*, external ventricular drain; *LD*, lumbar drain

**Table 4:** Distribution of shunt revisions of pediatric patients related to shunt system

Parameter	*n*	No revision	Revision	Univariate analysis (likelihood/Pearson)
		Total (%)	Total (%)	
	88	47 (53.4)	41 (46.6)	
Shunt system				0.0110/0.0122
proGAV	59	26 (44.1)	33 (55.9)	
proGAV 2.0	29	21 (72.4)	8 (27.6)

**Table 5:** Proportional-Hazards analysis of all shunt revisions in primary situation

Parameter	*n*	No revision	Revision	Odds ratio	95% confidence interval	Likelihood	Wald
		Total (%)	Total (%)				
Age							
Pediatric	88	47 (53.4)	41 (46.6)	2.752	− 0.6141/ − 0.1433	0.0018	0.0015
Elderly	118	97 (82.2)	21 (17.8)	0.312	− 0.3312/0.2089	0.6229	0.6206
Etiology							
IVH	24	13 (54.2)	11 (45.8)	0.206	− 0.4412/0.2393	0.4880	0.4781

*IVH*, intraventricular hemorrhage

### Revision rate

Data on revision surgeries were available for all patients. When focusing on age groups, our multivariate analysis revealed a significantly higher number of revisions in pediatric patients with 41 out of 88 patients (46.6%, Fisher’s exact test: *p* = 0.0093; Table 3). Concerning the different valve types, univariate analysis showed that pediatric patients with an implanted proGAV valve had to face revision surgery significantly more often than pediatric patients with a proGAV 2.0 valve (55.9% vs. 27.6%, Likelihood-ratio: *p* = 0.0110; Table 4). In contrast, of the 118 elderly patients we analyzed, there were 21 patients going through revision surgery (17.8%). Nevertheless, looking at the whole study population in primary situation, we observed no significant differences between the two shunt valves (25.1% vs. 23.9%, Likelihood-ratio: *p* = 0.8069).

Surprisingly, no significant association of etiology of hydrocephalus and revision rates was observed. For this analysis, we included primary vs. secondary hydrocephalus, hydrocephalus malresorptivus vs. hydrocephalus occlusus, typical underlying diseases, and localization of the implanted ventricular catheter (Table 3). However, patients with prior diversion of cerebrospinal fluid by using an external ventricular drainage, lumbar drainage, or an Ommaya reservoir experienced a significantly higher number of revisions (Likelihood-ratio: *p* < 0.0001).

### One-year revision rate

Based on our analysis, pediatric patients required more often surgical revisions within the first year after primary shunt implantation when compared to other patients (Likelihood-ratio: *p* < 0.0001, Fisher’s exact test: *p* = 0.0085). Notwithstanding, by looking at the two valve types in the pediatric group, we found a trend towards less revisions for the proGAV 2.0 but no significant difference in 1-year revision rate (40.7% vs. 27.6%, Likelihood-ratio: *p* = 0.2247; Table 2). Regarding 1-year revision rate, we identified a significantly higher number of revisions in patients with a prior diversion of cerebrospinal fluid through an Ommaya reservoir (66.7% vs. 33.3%, Fisher’s exact test: *p* = 0.0422). Furthermore, and when compared to patients with a catheter implantation in the left lateral ventricle or bilateral ventricle implantation, implantation in the right lateral ventricle was also associated with a higher number of revisions within 1 year (23.0% vs. 14.3%/16.7%, Fisher’s exact test: *p* = 0.0219). In contrast, patients suffering from idiopathic intracranial hypertension had to face no revision within 1 year after primary surgery (Likelihood-ratio: *p* = 0.0288, Fisher’s exact test: *p* = 0.0353). Regarding another important etiology of hydrocephalus, intraventricular hemorrhage, there was no significant result (Likelihood-ratio: *p* = 0.0556, Fisher’s exact test: *p* = 0.2344).

Comparing the two shunt valves, based on all patients in the primary situation, there was no significant difference in 1-year revision rate (Likelihood-ratio: *p* = 0.9077). Like our results regarding lifetime revision rate, we also did not find any significant result related to primary vs. secondary hydrocephalus (22.4% vs. 20.7%, Likelihood-ratio: *p* = 0.7613) and hydrocephalus malresorptivus vs. hydrocephalus occlusus (24.3% vs. 19.3%, Likelihood-ratio: *p* = 0.2538; Table 1).

### Revision-free survival

There was no significant difference in revision-free survival between the proGAV and proGAV 2.0 valve (mean 48.3 vs 25.9 months; Log-rank test: *p* = 0.6921). Pediatric patients faced a significantly shorter time of revision-free survival in our cohort (mean 36.1 vs 50.5 months; Log-rank test: *p* < 0.0001, Fisher’s exact test: *p* = 0.0093). Nevertheless, there was so significant difference in revision-free survival between the two shunt valves in our pediatric cohort (mean 32.7 vs 7.7 months; Log-rank test: *p* = 0.1381). For elderly patients, we did not observe a significant difference in revision-free survival when compared to all other patients (mean 27.7 vs 46.7 months; Log-rank test: *p* = 0.2394).

Although revision-free survival did not differ between patients with subarachnoid hemorrhage (mean 49.7 vs 46.4 months; Log-rank test: *p* = 0.4496), normal pressure hydrocephalus (mean 26.1 vs 47.7 months; Log-rank test: *p* = 0.7991), idiopathic intracranial hypertension (mean 38.9 vs 47.6 months; Log-rank test: *p* = 0.3259), and glioblastoma (mean 49.7 vs 46.4 months; Log-rank test: *p* = 0.7810).

Regarding prior diversion of cerebrospinal fluid, revision-free survival was significantly shorter in patients with prior diversion through Ommaya reservoir (mean 20.5 months; Log-rank test: *p* < 0.0001) compared to patients without prior diversion (mean 40.2 months) or diversion through an external ventricular drain (mean 48.6 months).

## Discussion

In our monocentric retrospective study, we compared two different shunt systems of the same manufacturer, the older valve type proGAV and the newer proGAV 2.0. Our focus was shunt failure in association with shunt valves and other potential and known risk factors for shunt failure.

### Valves

When looking at the whole study population, we were able to demonstrate that there is no significant difference between the two shunt valves regarding lifetime revision rate, revision-free survival, and overall survival. This observation might be explained by the similar mechanical construction of the gravitational unit of the two devices. Although the mechanical construction of the adjustable differential pressure unit of the two devices is different, allowing higher adjustment comfort, and reliability of the proGAV valve has already been proven in previous studies, we could find a slightly lower lifetime revision rate in patients with an implanted proGAV 2.0 valve (Table 3) [30]. However, this is statistically not significant and might also be explained by shorter follow-up of these patients. We clearly put emphasis on analyzing results regarding 1-year revision rate, since there is more chance for failure, due to a longer follow-up, in the proGAV group. Nevertheless, and almost surprisingly, we found no significant difference concerning 1-year revision rates between the two valves. To our knowledge, this is the first study investigating those two systems.

**Fig. 1:**
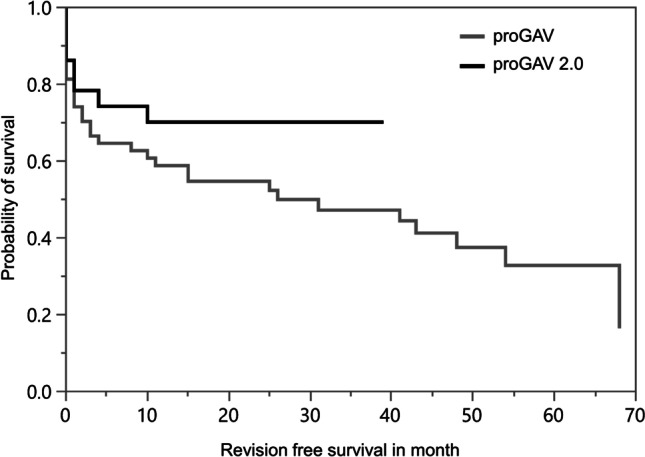
Revision-free survival for the different shunt valves in the primary situation

Although we found a significantly lower lifetime revision rate in pediatric patients with the proGAV 2.0 valve, we were not able to show this significant difference regarding 1-year revision rate (Table 1, Table 2, Fig. 1). Concerning the fact that we analyzed all shunt implantations from 2014 until 2020 and shunt implantation of the newer proGAV 2.0 valve started in 2017, there is a non-negligible longer follow-up in the proGAV-group. Taking this into account, 1-year revision rates should be seen as the more representative target variable when comparing the two shunt valves. Anderson et al. and other previous studies failed to show significant differences when comparing different valve types. In line with the literature, and due to similar mechanical construction of the two devices we analyzed, it is conceivably not surprising that we found no significant difference as well [4, 10, 19, 36].

### Patient age

Patient age was a key risk factor for shunt revisions. Previous studies already demonstrated revision rates as high as 40% in pediatric patients [4, 5, 31]. With 46.6% of all pediatric patients undergoing revision surgery within follow-up and a 36.4% 1-year revision rate, we found more or less similar results. Furthermore, in 2019 and 2020, Anderson et al. and Hauptmann et al. described an early shunt failure in revision cases [4, 13], which we observed in pediatric patients as well. In the secondary situation, 51% of all patients at our institution underwent revision surgery, 76.9% within 1 year.

Similar to findings of previous studies, we observed that besides the etiology of hydrocephalus, the presence of multiple shunt revisions in patient history, and prior CSF diversion, patient age plays one of the most significant role regarding shunt failure [13, 23, 27, 32, 33]. This seems to be of major relevance and overshadows the effects of changes in the shunt systems. For pediatric patients, Brunner et al. already compared the proGAV and proGAV2.0 valve in 262 pediatric patients [9]. In contrast to our cohort, the authors could show that the proGAV2.0 requires more often revisions in a short follow-up. In our cohort, the 1-year revision rate was lower for proGAV2.0 valve in 88 pediatric patients however without reaching significance most likely to the low patient number.

Interestingly, only 17.8% of elderly patients received revision surgery, 95% of the revision occurred within the 1st year after ventriculo-peritoneal shunt implantation. In univariate analysis, this proved to be significant, although in multivariate analysis we could not show a significant difference between elderly patients and the whole study population. Although there are several studies that have focused on shunt failure in pediatric and adult patients, there are only a few addressing elderly patients. Future studies should focus more on dividing the adult patient group allowing to gain further knowledge of this scientifically underrepresented age group. This seems to be even more of importance since we live in an aging population.

### Etiology of hydrocephalus

In our study, there was a trend in univariate analysis that some underlying diseases for hydrocephalus are associated with a higher 1-year revision rate, but except for idiopathic intracranial hypertension (with a significantly lower revision rate), our study did not show any relevant effects of etiology in multivariate analysis. There are numerous studies reporting on a significant association of etiology of hydrocephalus and shunt failure [13, 23]. Looking at pediatric patients, IVH as underlying disease has played an important role in previous scientific research and seems to be associated with a higher number of revision surgeries in patients obtaining shunt implantation [21, 24, 28, 29]. This observation is in line with our findings of a higher revision rate, higher 1-year revision rate, and lower revision-free survival in our univariate analysis for patients with IVH. Nevertheless, significance was not reached in multivariate analysis.

Greener et al. reported about higher failure rates of shunt devices and requirement of more frequent revisions in patients with IIH [12]. Surprisingly, we found a significantly lower 1-year revision rate in patients with IIH in our multivariate analysis (Table 1).

Although this might deliver knowledge concerning etiology of hydrocephalus and patient outcome after shunt implementation, origin of hydrocephalus seems to stay an unalterable risk factor.

### Catheter position

Numerous previous studies identified an association between accuracy of catheter placement and shunt survival [8, 16, 37] which is not very surprising, since wrong catheter placement needs revision surgery in some cases.

In the primary situation, we observed that shunt failure was caused by misplacement or migration of the proximal catheter in 13.1% of all revision cases, representing the fifth most frequent reason for revision surgery after shunt infection, catheter obstruction, distal catheter misplacement, and valve obstruction or malfunction. Likewise, Hauptmann et al. stated shunt obstruction and infection as their most frequent reasons for shunt failure [13].

In our multivariate analysis, we observed that implantation of the catheter in the right lateral ventricle was associated with a higher number of revisions within 1 year. However, we would not suggest taking this into account to change daily clinical routine since the normally non-dominant side is the right side with lower risk to relevant functional damage. Furthermore, there was a large difference in group size (left *n* = 84, right *n* = 304, bilateral *n* = 12). This also limits the interpretation of this finding.

### Prior CSF diversion

Our data show that prior CSF diversion, especially through an Ommaya reservoir, was associated with a higher revision rate and lower revision-free survival. About 77.8% of pediatric patients receiving an Ommaya reservoir prior to implantation of a ventriculoperitoneal shunt system needed revision surgery, of those 42.9% because of shunt infection. In contrast, 36.8% of shunt revisions in patients with prior implantation of an EVD were due to shunt infection. Interestingly, our data align with results of Orrego-González et al. and Reddy et al. [24, 25]. It might be useful to examine the association between prior implanted Ommaya reservoirs and infection of ventriculoperitoneal shunt systems in future studies. Since all Ommaya reservoirs had at least some but mostly multiple transdermal punctions before changing it into a shunt, it is rather likely that some kind of subclinical infection already existed before shunt operation.

## Conclusions

According to our data, besides lifetime risk of revision in pediatric patients, there is no significant difference between the proGAV and the proGAV 2.0. Risk factors for shunt failure such as patient age, etiology of hydrocephalus, association between prior diversion of CSF and shunt infection, and possible preventative measures are aspects that should clearly be part of future medical research.

## Limitations

Our observation of all shunt implantations in our institution from 2014 to 2020 comprises an almost twice as long follow-up in the proGAV-group than in the proGAV 2.0-group. This major limitation needs to be kept in mind when analyzing target variables such as lifetime revision rate and overall survival.

Furthermore, the number of patients regarding some of the etiologies of hydrocephalus like glioblastoma or idiopathic intracranial hemorrhage in our study was relatively small (Table 1, Table 3).

## Data Availability

All data and slides are still available on request.
